# Basic Emotions or Constructed Emotions: Insights From Taking an Evolutionary Perspective

**DOI:** 10.1177/17456916231205186

**Published:** 2023-11-02

**Authors:** Karlijn van Heijst, Mariska E. Kret, Annemie Ploeger

**Affiliations:** 1 Institute for Interdisciplinary Studies, University of Amsterdam; 2 Cognitive Psychology Unit, Faculty of Social and Behavioral Sciences, Leiden University; 3 Comparative Psychology and Affective Neuroscience Lab, Cognitive Psychology Department, Leiden University; 4 Leiden Institute for Brain and Cognition (LIBC), Leiden University; 5 Department of Psychology, University of Amsterdam

**Keywords:** basic emotion theory, theory of constructed emotion, emotion, feeling, evolution

## Abstract

The ongoing debate between basic emotion theories (BETs) and the theory of constructed emotion (TCE) hampers progress in the field of emotion research. Providing a new perspective, here we aim to bring the theories closer together by dissecting them according to Tinbergen’s four questions to clarify a focus on their evolutionary basis. On the basis of our review of the literature, we conclude that whereas BETs focus on the evolution question of Tinbergen, the TCE is more concerned with the causation of emotion. On the survival value of emotions both theories largely agree: to provide the best reaction in specific situations. Evidence is converging on the evolutionary history of emotions but is still limited for both theories—research within both frameworks focuses heavily on the causation. We conclude that BETs and the TCE explain two different phenomena: emotion and feeling. Therefore, they seem irreconcilable but possibly supplementary for explaining and investigating the evolution of emotion—especially considering their similar answer to the question of survival value. Last, this article further highlights the importance of carefully describing what aspect of emotion is being discussed or studied. Only then can evidence be interpreted to converge toward explaining emotion.

An ongoing debate in emotion research about the fundamental questions of what emotions exactly are and how they should be studied has occupied the minds of many researchers, with a recent revival (e.g., Adolphs, 2017; Barrett, 2017a; Frijda, 2016; Kret et al., 2022; LeDoux, 2020; Panksepp, 1998; Russell, 2003). This debate has implications for different aspects of emotion research: Are emotions social constructs or natural entities? Do emotions, as we experience them, have measurable neurophysiological correlates? Do they have distinct facial expressions? What is the value of the study of emotions in nonhuman animals? One commonality of these questions is that they still have no definite answers.

Of the many different theories on emotions, the two that have received the most attention are basic emotion theories (BETs) and the theory of constructed emotion (TCE). BETs state that there is a set of distinct emotions that are primitive and form the basis of all other (complex) emotions. Whether based on dedicated neurocircuitry (Panksepp, 1998; Panksepp & Watt, 2011), facial expressions (Ekman, 1992), function (Levenson, 2011), or a feeling component (Izard, 2011), the common thread of these theories is that the “basic” emotions are evolutionarily shaped because they served an adaptive advantage considering specific common threats. Basic emotions are different from other affective states and from each other in fundamental ways: They have, for example, distinctive universal signals, a distinctive physiology, and a dedicated function. There is consensus among theorists for at least five basic emotions: happiness, sadness, fear, anger, and disgust (for a review, see Tracy & Randles, 2011). Important early evidence consisted of studies on the universal recognition of facial expressions of emotions across cultures (Ekman, 1999; Ekman et al., 1987; Ekman & Friesen, 1971; for a review, see Keltner & Ekman, 2000). In the decades since then, however, BET researchers have broadened their focus to other expression modalities, as well as the social function of emotions (Keltner, Sauter, et al., 2019; Keltner, Tracy, et al., 2019).

The TCE was originally proposed in 2006 by Lisa Feldman Barrett (2006a, 2006b) and was formulated in its most complete form in 2017 (Barrett, 2017a, 2017b). Taking a different approach compared with basic emotion theorists, the TCE considers what a brain is for and is based on the concepts of interoception and allostasis. Interoception refers to the sensing of the internal state of the body, and allostasis is the process of maintaining and returning the body to homeostasis (Sterling, 2012). The TCE states that the primary function of the brain is to maintain allostasis and that emotions are consequences of this primary function. It combines evidence on macroscopic and microscopic connectivity patterns within the brain with computational theories of brain function and assumes that there is an allostatic-interoceptive system in the brain involving the limbic areas (Barrett & Simmons, 2015). In the TCE, emotion categories do not exist in nature independent of our perception of them. Instead, emotions are constructed concepts in the brain based on past experience and are related to the current state of the body and the environment (Barrett, 2006a). Therefore, specific emotion categories are not linked to specific facial expressions, physiology, antecedent events, and so on (Barrett, 2017a). Instead, the emotions humans experience are heavily dependent on the specific situations and the emotion concepts (individual) humans have formed.

It is necessary to underline that the TCE is broader than a theory of emotion per se. It is a more general theory of social construction, according to which all mental events are a consequence of how the brain coordinates bodily regulation (allostasis) and plans action accordingly (Shaffer et al., 2022). However, the theory does propose clear ideas on emotions that differ in major ways from BETs, continuing a long-standing debate in the literature (see below). Moreover, a considerable amount of the literature published within the TCE framework has focused on emotions. We therefore argue it is valuable to isolate the TCE’s views on emotions to compare them with BETs.

Although a constructionist approach to emotion has existed for well over a century and the debate between BETs and the TCE is by no means a recent development, furthering this debate continues to be relevant to move the field of emotion research forward. TCE researchers continue to heavily criticize BETs, and BET researchers in turn criticize the TCE (e.g., Gendron et al., 2015; Lench et al., 2011, 2013; Lindquist et al., 2013; Sauter et al., 2015). In the meantime, emotion scientists gather evidence in support of both theories or draw different conclusions in reviews and meta-analyses partly on the basis of the same evidence (e.g., Kirby & Robinson, 2017; Lindquist et al., 2012; Vytal & Hamann, 2009). This results in an interesting but confusing debate, which makes it challenging to draw general conclusions that can move the field forward and guide research for better treatment of emotion disorders.

The debate between BETs and the TCE reflects a similar debate that is going on within evolutionary biology and evolutionary psychology. A cornerstone of evolutionary biology is the modern synthesis, in which Darwin’s famous theory of evolution by natural selection was synthesized with knowledge gained by geneticists (Huxley, 1942). The modern synthesis has been extended with new findings related to epigenetics, niche construction, developmental plasticity, multilevel selection, and more—called the extended evolutionary synthesis (Laland et al., 2015).

Evolutionary psychology involves the study of behavior and the mind from an evolutionary perspective, fully embracing the modern synthesis (Tooby & Cosmides, 1990). It is has been argued that evolutionary psychology needs to include development at its core (e.g., Ploeger et al., 2008), or that it needs to change into developmental systems theory (Lickliter & Honeycutt, 2003) and adhere to the extended evolutionary synthesis (Narvaez et al., 2022). Developmental system theorists argue that organisms inherit a full developmental system—including genes, bodily structures, the direct environment, and the full ecology—and that natural selection acts on the developmental system as a whole and not on specific elements such as genes. In addition, natural selection is not regarded as the sole source of evolutionary change—it is argued that development itself, among other factors, leads to new evolutionary milestones.

Despite attempts to integrate evolutionary psychology and developmental systems theory (Bjorklund et al., 2007; Frankenhuis et al., 2013; Ploeger & Galis, 2011), the debate continues without much progress, with classic evolutionary psychologists arguing that their approach is revolutionary (Buss, 2020), developmental system theorists arguing that evolutionary psychology is completely wrong (Narvaez et al., 2022), evolutionary developmental psychologists trying to integrate the two positions (Bjorklund et al., 2022), and developmental systems theorists replying that it is their way or no way at all (Moore et al., 2022), thereby ignoring that the extended evolutionary synthesis is an extension of, not a replacement for, the classic modern synthesis. We argue that this is not a fruitful way toward scientific progress. As we have seen in most debates in science, such as the major nature–nurture debate, it is hardly ever one way or the other—to create a theory that covers the full spectrum of the phenomenon under consideration, we need to address both sides of the coin.

We argue that a similar kind of debate is going on between BETs and the TCE. BETs are grounded in the modern synthesis, with the emphasis on emotions as adaptations evolved by natural selection. The TCE relates to the extended evolutionary synthesis, with its emphasis on development and variation (Barrett, 2022; Barrett & Lida, in press). In addition, there is ample empirical evidence for both positions, just like there is ample evidence in evolutionary biology for both the modern synthesis and the extended evolutionary synthesis. It is not just one or the other, but these two approaches complement each other. In this article, we argue that both BETs and the TCE have their merits and need to be integrated to fully comprehend the evolution and development of emotions.

Many different attempts have already been made to bring BETs and the TCE closer together (e.g., Lange et al., 2020; Lewis & Liu, 2011; Moors, 2017; Scherer, 2022). For instance, Lange et al. (2020) proposed an integrative psychometric model of emotions, bridging the two theories. Taking a social-psychological approach, the authors introduced a novel psychometric network model that integrates affect program (including BET), constructionist (including the TCE), and appraisal (including BET) theories and allows for all the specific properties of emotions these theories explain. However, as of yet, no attempt has been made to dive deeper into their evolutionary basis. BETs are tightly linked to evolutionary theory, and Barrett also stated that her theory has evolutionary plausibility (Barrett, 2006b, 2017a, 2017b). Here, we aim to bring the theories closer together by taking an evolutionary perspective. First, we divide (research within) both theories by Tinbergen’s four questions (Tinbergen, 1963/2005; Box 1). Discussing selected literature in answer to the questions, we analyze the evolutionary basis of BETs and the TCE. Aiming to provide a bridge between the theories using Tinbergen’s last question, we have found that even at this deeper level that is challenging. Our review underlines the importance of semantics and clearly defining any aspect of emotion under discussion or study—especially emotion versus feeling. Moreover, we propose that the bridge between BETs and the TCE might instead lie with their similar answer to the question of the survival value of emotion: to provide an animal’s best response to promote survival and well-being in specific circumstances. We conclude that with this overlapping focus, both BETs and the TCE are important in moving the field of emotion science forward because they can provide answers to different questions about emotion.

**Box 1.** Tinbergen’s Four Questions

Tinbergen’s four questions can be helpful to make a distinction between the different levels—or different aspects—of emotions that can be studied (Tinbergen, 1963/2005): • *Causation* concerns a mechanistic explanation of behavior. In a publication marking the 50-year anniversary of Tinbergen’s publication, Bateson and Laland (2013) described how Tinbergen’s problem of causation concerns the “mechanism of control” and relates to the question “how does it work.” • *Ontogeny* concerns the answer to the question “how did it develop” (Bateson & Laland, 2013). • *Survival value* (or function) provides an answer to the question “what is it (good) for.” To emphasize the present-day focus of the question, Bateson and Laland (2013) proposed the term “current utility” for Tinbergen’s problem of survival value. • Tinbergen proposed that the study of *evolution* has two aims: “The elucidation of the course evolution must be assumed to have taken, and the unraveling of its dynamics” (Tinbergen, 1963/2005, p. 316). It concerns the question “how did it evolve” (Bateson & Laland, 2013), which implies that we need to study the evolutionary history of behavior, including the comparison of the behavior of related species.

## Tinbergen’s Question of Causation in the View of BETs and the TCE

To focus on the evolutionary basis of BETs and the TCE we follow a Tinbergian approach (see Box 1), which makes a distinction between four different types of questions that can be asked when studying a trait, in our case emotions. In addition to evolution, these include causation, function, and ontogeny.

Starting with a short overview of research on causation, in BETs different basic emotions or core reaction patterns are quick responses that have universals in antecedent events, physiological changes, involved neurocircuitry, expressions, behavior, and so on (for an overview of BETs, see Tracy & Randles, 2011). These core reaction patterns form the basis of the more complex emotions that humans from a certain age have. Evidence in support of the existence of basic emotions includes universal recognition (Ekman et al., 1987; Ekman & Friesen, 1971; Keltner & Ekman, 2000; Shuman et al., 2017) and production (Cordaro et al., 2018; Cowen et al., 2021) of facial expressions across cultures, as well as other modes of expression (Keltner, Sauter, et al., 2019). Whether studying recognition or production, however, this evidence is mostly based on posed or acted expressions of emotions. Evidence on production and recognition of *spontaneous* emotional expressions is mostly lacking (but see Naab & Russell, 2007), but there is some literature on spontaneous expressions of emotions in infants (see below). Facial expressions also play a significant role in human communication, which has been extensively studied in the literature. During social interactions, we often automatically mimic the emotional expressions of others, leading to a synchronization of internal states.

Researchers have used facial electromyographic techniques to investigate the physiological reactions that occur when people are exposed to emotional facial expressions. These studies have revealed that individuals spontaneously react with distinct facial-muscle responses that are relevant to the specific emotion being observed. These reactions can be seen as a form of mimicry because individuals tend to mirror the facial stimuli they encounter. A groundbreaking study by Dimberg et al. (2000) aimed to determine whether similar facial reactions could be elicited when individuals were unconsciously exposed to facial expressions. Using the backward-masking technique, the researchers prevented subjects from consciously perceiving 30-ms exposures of happy, neutral, and angry faces. The emotionally expressive faces were immediately followed and masked by neutral faces. Despite the subjects’ lack of conscious awareness of the happy and angry faces, they still exhibited distinct facial-muscle reactions corresponding to the emotional stimuli. This study demonstrated that both positive and negative emotional reactions can be unconsciously evoked, highlighting the existence of important aspects of emotional face-to-face communication that occur on an unconscious level.

Considering other aspects of causation for BETs, no specific pattern of autonomous nervous system (ANS) activity has been found for the broad categories of emotions, for example, anger, but studies do show specificity for subtypes of these categories (e.g., withdrawal-oriented anger or anger in self-defense; for a review, see Kreibig, 2010). Specific ANS activity might also play a role in emotion perception. In a recent study, researchers found a peak in skin conductance when human participants were passively viewing angry facial expressions, and a drop in skin temperature was associated with sad body expressions (Folz et al., 2022). Several meta-analyses of BET on imaging studies of emotion have shown that there are distinct regional brain activation patterns of different basic emotions, although there is an overlap of involved brain regions (see Kirby & Robinson, 2017; Vytal & Hamann, 2009). It is important to note, however, that there is considerable variation in the methods used during imaging studies. Most focus on the perception of emotions from, for example, facial expressions. If emotional states are induced, researchers have used a wide variety of methods, from mental imagery to showing videos with emotional content (e.g., see Benuzzi et al., 2008; Fitzgerald et al., 2006; Pelletier et al., 2003).

TCE researchers have provided a detailed description of the causation of emotion (and other mental events), including active inference, allostasis, interoception (Barrett & Simmons, 2015), *core affect* (Barrett & Bliss-Moreau, 2009), and the formation of concepts (Barrett, 2017a, 2017b). In a critique of BET, TCE researchers showed in a meta-analysis of neuroimaging studies of emotion that several different basic emotions (fear, disgust, anger, and sadness) are not specifically associated with activity in single brain regions (Lindquist et al., 2012; note, however, that this locationist search has now been abandoned by BET researchers). Studying brain activation in emotion within a TCE framework, researchers have shown intrinsic connectivity between regions of the proposed interoceptive system by performing functional connectivity analyses on resting-state functional MRI (fMRI) scans of human participants (Kleckner et al., 2017). A different study on the interoceptive system showed increased activity in the primary interoceptive cortex (Barrett & Simmons, 2015) when human participants were imagining experiences that involved heightened interoceptive sensations (Wilson-Mendenhall et al., 2019).

Emotion words have a central role in the TCE because they are a means of labeling experiences (Barrett, 2017a). Focusing on the role of language and culture in the recognition of emotions from facial expressions, several studies have shown that American participants label facial expressions of emotions with basic emotion words more frequently than people from a remote “traditional” or hunter-gatherer society. When sorting expressions into piles, American participants’ piles also mapped better onto discrete emotion categories proposed by Western researchers (Gendron et al., 2014, 2020). However, another study did not support the importance of emotion words (Hoemann et al., 2022). American adults had to learn three new emotion categories that do not have a specific linguistic label in the United States but do in Japan, Thailand, and Germany. The participants were able to learn these new emotion categories, but providing emotion labels as feedback did not facilitate learning.

Most empirical evidence to date is concerned with the question of causation of emotions, both for BETs and the TCE, although with a different focus. In addition to a large body of literature on facial expressions of emotions, research within the BET framework has also focused on other expression modalities and on ANS and central nervous system activity. The main focus has been a limited set of basic emotions, but in recent years the focus has broadened and become more nuanced. In the TCE, research on emotions that falls within causation explicitly does not have a focus on emotion categories, but on brain mechanisms of interoception and the role of emotion concepts/words.

## Tinbergen’s Question of Ontogeny in the View of BETs and the TCE

Considering the question of ontogeny, BETs make the case for a large innate component in basic emotions and therefore that it can be assumed that (very young) infants show and recognize the same emotions—at least in some very basic form. However, BETs also allow for development and culture to influence variations around the core characteristics of the different basic emotions (Ekman, 1992; Ekman & Cordaro, 2011; Izard, 2007, 2011; Levenson, 2011; Panksepp & Watt, 2011). Research in infants has generally focused on facial expressions of emotions. Early studies on the production of facial expressions showed that newborn infants imitate mouth openings and tongue protrusions (Meltzoff & Moore, 1983) and gape aversively in response to bitter tastes (Berridge, 2000; Steiner et al., 2001). Considering recognizing facial-emotion expression, a classic study revealed that newborns can discriminate between surprised, happy, and sad faces (Field et al., 1982). The newborns even reliably copied the expressions, as was judged by adults blind to the condition. A meta-analysis on 336 effect sizes revealed significant evidence for neonatal imitation (not just facial expressions), although there was substantial variation in the results (Davis et al., 2021). In general, it can be concluded that young infants can discriminate between positive and negative facial expressions of emotion. However, only few studies have examined this, and results vary greatly depending on the methodology used (Ruba & Repacholi, 2019).

TCE researchers have provided a detailed account of emotional development in which language development plays a crucial role (Barrett, 2017a; Hoemann et al., 2019, 2020). Infants need to infer similarities among patterns (e.g., the expression of fear) to learn an abstract concept. This process is very similar to learning about objects, and so learning about emotion categories is not domain-specific but follows general patterns of learning (Hoemann et al., 2020). As far as we know, there are no empirical studies with infants testing the TCE.

## Tinbergen’s Question of Survival Value in the View of BETs and the TCE

Experimental evidence on the direct function (survival value) of emotions is mostly lacking, perhaps because it seems so straightforward. Moreover, following a Tinbergian approach, gathering the most convincing evidence—direct evidence of the survival value of emotions— would be challenging and involve ethical constraints. An idea for a convincing study of the direct survival value of fear could, for example, involve releasing a pride of hungry lions in the middle of a square full of people. A more realistic but also more indirect method can focus on differences between closely related species and how these have adapted differently to their specific environments (Tinbergen, 1963/2005).

Foundational to BETs is their proposed function of basic emotions, which is rooted in their evolutionary basis: They prepare the animal to react quickly and adequately to (interpersonal) fundamental life events (Ekman, 1992; Ekman & Cordaro, 2011; Izard, 2007, 2011; Levenson, 2011). Following that emotions are accompanied by bodily and behavioral reactions that are helpful in a certain situation, a direct function of, for example, ANS activity and facial expressions of emotions has also been proposed (Ekman & Cordaro, 2011; Kreibig, 2010; Lee et al., 2013; Susskind et al., 2008). Furthermore, it has been argued that emotional expressions have an important social function and may thus have been especially of survival value in social species (e.g., see Niedenthal & Brauer, 2011; van Kleef et al., 2016). The function of basic emotions can be most easily observed in infants or extreme situations in adults. However, as cognitive abilities increase, individuals will be better able to regulate emotions, and primitive reactions will serve more to change probability (Ekman & Cordaro, 2011; Izard, 2011; Levenson, 2011; Panksepp & Watt, 2011). It is important to note here that the function of emotions is rarely discussed under BETs as “current utility” (i.e., what purpose they serve in the present day), a distinction that is made in Tinbergen’s four questions (Tinbergen, 1963/2005; Box 1)—although BET theorists agree that what was adaptive in the past does not always serve a function in the same situations in the present.

When it comes to the current utility or function of emotions, the TCE arguably does not differ much from BETs: In the TCE emotion(s) (concepts) also are affective states that occur in specific situations—in the TCE specifically including internal sensations—and guide appropriate action to promote surviving and thriving of the animal (Barrett, 2017a). However, in the TCE the current utility of emotions is only an example of the current utility of all mental events, which are a consequence of the more general function of the brain. Indeed, within the TCE the abovementioned current utility of emotions is the current utility of brain structure and function in general (Barrett, 2017a, 2017b; Shaffer et al., 2022). Therefore, within the TCE the study of the current utility of emotions would not focus on specific emotion categories but on the more general current utility of active inference, interoception (recently discussed by Quigley et al., 2021), core affective experience, the formation of concepts, and then, further on, mental, goal-based categories. Barrett (2017a) listed a few other functions of emotion concepts specifically: to make meaning of one’s sensations and actions, regulation of the body budget (allostasis), emotion communication, and social influence (influencing other people’s body budgets/allostasis). Arguably, the TCE has a larger focus on the current utility of emotions in contrast with function throughout evolution.

## Tinbergen’s Question of Evolutionary History in the View of BETs and the TCE

### Evolutionary history in BETs

BETs hypothesize that emotions are direct adaptations—that is, that they were shaped by evolution by natural selection (Ekman & Cordaro, 2011; Izard, 2011; Levenson, 2011; Panksepp & Watt, 2011). To study the course of evolution, we can examine the emotions of our closest living relatives, great apes, and other animals by studying their expressions, cognitive biases, preferences, and so forth.

Expressions of emotions in animals have started to receive more attention in recent years, but early descriptions of expressions in great apes date back to almost a century ago (e.g., de Waal, 1988; Ladygina-Kohts, 1935/2002; van Hooff, 1972; van Lawick-Goodall, 1968). For a review on the production of expressions of emotions in human and nonhuman great apes, see Kret et al. (2020). The facial expression of the basic emotion category fear shows considerable overlap between humans and great apes. Furthermore, the human smile and facial expression accompanying laughter have clear suspected homologues in the expressions of great apes: the silent-bared-teeth face and play face. The latter is also present in other mammals and likely evolved in the context of play to serve a social function (Davila-Ross & Palagi, 2022; Palagi et al., 2022). For anger/aggression, surprise, and sadness, there is so far no evidence of clear facial expressions in great apes—although that does not necessarily imply that they do not exist and that they are not similar. A classic example of similarities in facial expressions of emotion is the seminal study by Steiner et al. (2001) on the reactions to different tastes of human infants and 11 primate species (great apes, Old World and New World monkeys). The researchers measured affective reactions to fluids that tasted sweet, sour, bitter, and neutral (water). The human infants and all 11 other primate species showed (nearly) universal components of facial expressions to sweet and bitter tastes.

When it comes to bodily expressions, which possibly play a more important role in great apes compared with humans, there is some clear overlap for anger and aggression between all great ape species and humans, but there are also differences. In chimpanzees, bodily expressions of fear/anxiety (crouching, making oneself small, scratching) and affiliation/positive affect (expressions of wrestling chimpanzees vs. humans being tickled) show similarities with those in humans. For these emotion categories, a systematic analysis in other great ape species is lacking. Bodily expressions of disgust/aversion and sadness/grief have not been systematically studied in any of the great ape species (Kret et al., 2020). Linking to the previous section, a study focusing on body expressions of emotion in chimpanzees has also given insight into the possible survival value. In a group of chimpanzees Menzel (1974) found that when he introduced a toy snake, individuals that had not seen it adopted the same posture as the individual that had, showing the value of emotion communication.

Many studies have focused on vocalizations in great apes for fear and anxiety, mostly in naturalistic settings. There is some overlap with human fear screams, but these are usually studied with artificially produced vocal stimuli (by actors). For laughter as an expression of affiliation/positive affect, there is clear overlap in form and function between humans and great apes, and the same goes for threat and aggression (Davila-Ross & Palagi, 2022; Kret et al., 2020). Evidence for the possible evolutionarily older origins of laughter come from the seminal work of Jaak Panksepp. Studying high-frequency vocalizations in response to tickling in rats, Panksepp and Burgdorf (1999) found that, like human laughter, there is rapid conditioning of this tickling response, it is stronger for certain body parts, and the high-frequency vocalizations occur naturally during play and are reduced in stressful situations.

In addition to the study on laughter in rats, Panksepp’s work includes comparative evidence for the neural circuitry and neurochemistry of emotions in animals (again mostly focusing on rodents and not including great apes; Panksepp, 2005, 2007). Following his version of BET, animals show seeking behavior when the SEEKING circuit is stimulated directly. Dopamine, which is involved in this circuit, has been shown to be euphoric in humans. For PANIC (or separation distress), research has shown that certain neurotransmitters or drugs alleviate or intensify the behavior associated with separation distress in different animal species. Furthermore, stimulation of certain subcortical areas can provoke separation cries in animals, and imaging studies have shown that similar trajectories of brain activation are involved/activated in humans experiencing sadness (Panksepp, 2005, 2007). In FEAR research, there is evidence for several different brain systems orchestrating fear responses, all including the amygdala (e.g., Ledoux, 2003).

Last, some studies have focused on ANS activity related to emotions in apes. Parr (2001) showed that peripheral skin temperature in chimpanzees decreased more when viewing videos of conspecifics being injected with needles, or videos of darts and needles alone, compared with viewing conspecifics showing agonism directed toward veterinarians. Indicative of negative sympathetic arousal, these decreases in skin temperature suggest chimpanzees were in a fear state in response to viewing darts and needles. Furthermore, they indicate possible affective sharing in response to conspecifics’ fear or pain.

### Evolutionary history in the TCE

Unraveling the course of evolution in the TCE, one would have to study the more general brain processes of active inference, interoception (core affect), and the formation of concepts in animals—especially great apes, but certain bird species (e.g., ravens) could also be an interesting avenue for research. In the TCE, it is assumed that the proposed interoceptive system is also present in the brains of animals (especially great apes and other mammals), which provides an opportunity to study it. Furthermore, the TCE does support the view that most animals (again, especially mammals and great apes in particular) can experience some form of affect and can create some concepts. A second study reported in Parr (2001) indeed suggests that chimpanzees can infer positive versus negative valence from facial expressions of conspecifics. Without prior training, subjects spontaneously matched emotional videos to conspecific facial expressions on the basis of their valence. However, Barrett (2017a) argued that there is no convincing evidence yet that animals can also create mental, goal-based concepts, such as emotions. Of note, some findings challenge this position, for example, the classic study by Brosnan and de Waal (2003) that showed capuchin monkeys might have a sense of fairness. Moreover, because in the TCE emotion concepts are tightly linked to language, it places a lot of doubt on whether animals can experience the same emotions humans do. This implicates that “our” emotion concepts cannot be directly studied in animals in the way BETs propose (Barrett, 2017a).

Several studies inspired by the TCE have investigated (core) affect in rhesus macaques (*Macaca mulatta*). One study showed that when passively viewing 30-s social videos that changed from negative to positive valence, sympathetic ANS activity in monkeys decreased and parasympathetic ANS activity increased, respectively (Bliss-Moreau et al., 2013). Similar patterns of ANS activity have been reported in humans in response to affective stimuli, suggesting converging evolution (for a review, see, e.g., Mendes, 2009). Furthermore, a resting-state fMRI study has shown that the ventral and to a lesser extent the dorsal subnetworks of the salience network in rhesus macaques show overlap with the networks that have been found in humans (Touroutoglou et al., 2016). Assuming an important role for the salience network in allostasis and core affect, these results suggest similar (basic) circuitry might be responsible for affect in humans and its proposed presence in all mammals. A last study showed that while viewing aggressive or submissive videos of a single conspecific, monkeys fixated longer and more often on conspecifics bodies than their heads. Furthermore, subjects fixated first on bodies in the videos depicting aggressive and submissive affective behaviors (Bliss-Moreau et al., 2017). These results show that, as in humans, information about the body might be important for rhesus macaques when processing emotions, suggesting the role of body expressions of emotions might be evolutionarily old.

### Conclusion

In conclusion, there is converging evidence for the homology of certain (aspects) of expressions of various emotion categories in nonhuman primates, in line with BETs. There is also some evidence on shared brain circuitry of specific basic emotion categories and for similarities in ANS activity between chimpanzees and humans. In the TCE, the study of evolutionary history has so far focused on ANS activity, core affect, and attentional bias in rhesus macaques. Overall, evidence is still limited for the evolutionary history of emotions within both theories.

## Discussion: Evolution of Emotion or Evolution of Feeling?

BETs and the TCE are very different theories, and this is no less true when it comes to their evolutionary basis. The evidence discussed above highlights the different focus of research within the BETs and TCE frameworks. When dividing the evidence by Tinbergen’s four questions (Tinbergen, 1963/2005), it becomes clear that for both theories most evidence has been gathered on the causation of emotions, albeit with a different focus: Within BETs the focus has been on defining different basic emotion categories and their characteristics, whereas the TCE has focused on defining the brain circuitry and mechanisms that are at the basis of the formation of emotion concepts (and other mental events). Some work has been done on ontogeny within the BET framework, but convincing evidence is lacking, and for the TCE no empirical studies on ontogeny are available yet. Similarly, there is limited evidence on the current survival value of emotions, although the theories propose similar answers to this question: to guide appropriate action in specific situations to increase survival and well-being of the individual. When it comes to studying the evolutionary history, there also is limited evidence on both sides. Taking a step back from the evidence, and considering the theories themselves, we propose that BETs focus on Tinbergen’s evolution question, whereas the TCE focuses on causation.

Considering these many differences, how then can we bring BETs and the TCE together? Perhaps we need to dig deeper and start with what each theory is exactly about and what exactly each theory tries to explain. We argue that BETs focus on primary, basic, but specific bioregulatory states of the body in reaction to certain basic (or extreme) events that have been shaped by evolution to be adaptive and promote survival in these situations (see Ekman & Cordaro, 2011; Izard, 2011; Levenson, 2011; Panksepp & Watt, 2011). These states are accompanied by subjective experiences, but BETS do not focus on these. Conversely, the TCE aims to explain (all) experiences, all mental events, and how they arise. Specifically applied to emotions, the TCE thus aims to explain the subjective, conscious experiences of emotions that humans have that can be considered feelings. Of note, experience in the TCE is tightly coupled to bodily regulation and action planning, so the theory also accounts for physiological states. However, applied to emotions, these are building blocks of subjective emotional experiences (or those of others) and the labels we use for these experiences. Thus, we propose that BETs are theories of emotion and that the TCE is a theory of feeling (although for Barrett’s opposition to this view, see Adolphs et al., 2019; for definitions used, see Box 2). The same distinction has been made before by De Waal (2019, p. 256) for the debate between Barrett and Panksepp. From the literature, we further conclude that in the TCE the term emotion is at times applied beyond feeling, describing higher cognitive processes involved with explicit subjective awareness of emotion states (Barrett, 2017a). This can make it challenging to interpret and compare the theory because it is not always clear what level of consciousness is implied. For the current purpose we discuss the distinction between a focus on emotion in BETs and on feeling in the TCE.

**Box 2.** Definitions

*Emotion.* Following Damasio (2004), in this article we use the definition of emotion as a bioregulatory response to an external event that promotes survival. More specifically, these bioregulatory responses promote physiological states that secure the survival and well-being of the individual. These responses can be studied in human and nonhuman animals. *Feeling.* We use the definition of feeling as “the *mental representation* of the physiological changes that occur during an emotion” (Damasio, 2004, p. 52). Mental representation can also be defined as the *cognitive interpretation* of the physiological changes. Feelings can arguably be studied only in humans and can be measured only indirectly (by verbal/written report). *Core affect.* Core affect combines valence, which describes pleasure versus displeasure, and arousal, which describes arousal/activation versus calmness (Russell, 1980, 2003). Valence and arousal can be mapped onto axes that when combined form a graph with four quadrants describing a core affective state (Russell, 1980). Note that in using this definition core affect is a *feeling* state. Core affect is referred to by theory of constructed emotion researchers and others as the psychological primitive of emotion and can be experienced by human and nonhuman animals. Note, however, that Shaffer et al. (2022) proposed it is a more general property of consciousness, not specific to emotions.

An area of research that is of special interest for this proposed distinction between emotion in BETs and feeling in the TCE is human empathy and how humans react to the emotions of others. Even without conscious awareness, the emotional expressions displayed by others capture our immediate attention and have the power to influence our own emotions. Interestingly, how they influence our bioregulatory states (emotions) and our subjective experience (feeling) may not always align. Following the study by Dimberg et al. (2000) discussed above, subsequent research discovered that mimicry has profound consequences for the course of interpersonal interactions. Individuals who were mimicked by their interaction partners were found to be more likable, trustworthy, attractive, and received more empathic reactions (e.g., Chartrand & van Baaren, 2009). These positive consequences have also been observed even when the mimicry occurred on a completely unconscious and uncontrollable level, such as with pupil mimicry (Kret et al., 2015) or physiological synchrony (e.g., Behrens et al., 2020; Galbusera et al., 2019; Levenson & Gottman, 1983; Prochazkova et al., 2022; for a review, see Prochazkova and Kret, 2017). These findings suggest that human empathy is often automated to a large extent (de Waal & Preston, 2017).

To gain a deeper understanding of how humans perceive the emotions of others without asking them directly, experimental psychologists have designed various implicit experimental paradigms. Examples include dot-probe tests, emotional Stroop tasks, flanker tasks, or implicit-association tests, which can reveal biases in cognition or attention or positive or negative associations that people have with, for example, Black versus White faces (Greenwald & Banaji, 1995). Techniques such as eye-tracking, psychophysiological, and neural measurements give further insights into these processes that are from the outside often invisible. By bypassing the language filters, which can introduce cultural biases, these implicit measures provide more direct information about individuals’ interpretations of emotional cues from others. It is proposed that these reactions are likely to be less influenced by cultural factors, indicating that our physiological and neural responses to emotions (the focus of BETs) may not always align with how we construct and articulate them (which is the focus of the TCE). This discrepancy in our perception of the emotions of others—between our bioregulatory states and our subjective experience in response to them—is food for an interesting discussion of how the way we discuss our feelings is at the same time an interpretation.

Making the distinction between emotions as bioregulatory states of the body and feelings as subjective experiences when explaining the evolutionary basis of emotion, BETs and the TCE are trying to explain two different traits—or rather combinations of traits. Arguably, the main focus of BETs is on what can be observed about emotions: behavior, expressions, (neuro)physiology, and emotion recognition. BETs seem in this sense strongly inspired by Darwin’s own writings on emotions (Darwin, 1872/2009) and not so concerned with feelings (ca. Izard, 2011). Importantly, apart from a specific set of several distinct, basic, and adaptive responses, BETs do not claim anything specific about all other emotions or feelings—although they do put forward frameworks in which these basic emotions are building blocks of other, more complex emotions and moods.

The evolutionary basis of feeling, as we propose is the focus of the TCE applied to emotions, is more complicated. Feeling is subjective and exists only in the brain of the individual experiencing it, so there cannot be an evolutionary basis to specific subjective experiences of emotion. What can be studied with regard to feeling and higher cognitive subjective experiences of emotion is how they arise in the brain and how evolution influenced this process. The TCE hypothesizes that the subjective experience of emotion is a result of allostasis, interoception, and active inference. These building blocks aside, the study of feeling is heavily tied to the study of language, cognition, and consciousness—and therefore to the study of the evolution of these phenomena. It is arguably too challenging to provide evidence of evolution by natural selection for cognition and thus for any mental event (for a critique on the study of evolution of cognition, see, e.g., Lewontin, 1990). It might, however, be possible to track the building blocks of emotional experience in other animals, thus tracing the evolutionary history. These building blocks are interoception, experiencing core affective feelings, and creating concepts. As discussed above, TCE researchers have provided some evidence for a core interoceptive network in rhesus macaques, and many studies have shown that animals, especially primates and certain bird species, can create concepts (see, e.g., Wright et al., 2021). Furthermore, in the TCE it is assumed that animals can experience core affective feelings (for a definition of core affect, see Box 2; Barrett, 2017a; Bliss-Moreau, 2017). However, when discussing animals experiencing core affective feelings, the same issues arguably arise as when discussing animals experiencing, for example, fear (for a similar point of view, see Viola, 2017). Views on animal subjective experience of emotion and affect are diverse, and a detailed discussion is beyond the scope of this article (although for a recent opinion article, see Kret et al., 2022). We can arguably approach animal subjective feeling by using carefully designed tasks (e.g., assessing cognitive bias), but we can never know their experience exactly because we cannot ask them.

The discussion about the differences between the study of evolution of emotion in BETs and evolution of feeling in the TCE and its implications aside, what is at the heart of this discussion is assessing what about emotions was adaptively relevant throughout evolution for survival and reproduction. When discussing the adaptive value of emotional behaviors, one might need to look at more basic adaptive behaviors instead of focusing on words/categories. One could make the case that the function of a specific trait is the most important and that emotions can be categorized accordingly (e.g., Adolphs, 2017; Sznycer et al., 2017; Tooby & Cosmides, 2008). Another point of view is that valence and arousal are the basic adaptive properties of emotions. Indeed, it has been argued by different theorists, Barrett included, that valence and arousal have been shaped by evolution and are adaptive (e.g., Barrett & Bliss-Moreau, 2009; Mendl et al., 2010; Mendl & Paul, 2020; Russell, 2003)—although a conscious experience/feeling aspect is not always implied—and it has been suggested that all animals possess valence and arousal to a certain extent (Bliss-Moreau, 2017).

The reviewed literature underlines that semantics and careful and precise use of definitions are crucial in emotion research and theories. It is important to make clear what exactly about emotion is being investigated or discussed. Starting at a broad level, one needs to assess what aspects of emotion there are. This list can arguably include the emotion (the bioregulatory response and its physiological manifestation in the body), the behavior that is caused by it, expressions of emotions (facial, bodily, vocal), the subjective experience of emotion (feeling), and the social aspects: recognition of emotional behavior or expressions, feelings about the emotional expressions of others, and metacognitive processes involving emotion (e.g., reflection on feelings). Only by carefully defining what aspect is under study will we be able to understand the evolutionary basis of all different traits that are generally discussed under the term emotion. Specifically considering the evolutionary history, when studying animal emotions it is helpful to explicitly consider how inferences are being made from the study of human emotions (for a recent opinion article, see Mendl et al., 2022). For example, the use of emotional experience/mental labels to distinguish between different physiological responses in animals might be confusing because we cannot know what label (if any) of feeling belongs to it.

## Conclusion

Comparing two main (groups of) emotion theories on their evolutionary basis, we have found that even at this deeper level, it is a challenge to find common ground between BETs and the TCE (when applied to emotions). It occurs to us that the two theories focus on different aspects of what broadly fall under the label emotion: emotion and feeling. Furthermore, the theories also have a different focus when it comes to which of Tinbergen’s questions they aim to provide an answer to: the evolution question of emotion in BETs and the causation of feeling in the TCE. This makes their focus even more distinct from each other. Scrutinizing the seemingly clashing evolutionary basis of both theories clearly underlines how important it is to make distinctions between different aspects of emotion studied. The pertinent question is what exactly is the trait of which the evolutionary basis is under study. Emotion is an ill-defined and complex trait, and we are at the scientific basis of understanding what aspects of it were adaptively relevant in our evolutionary past and what this complex trait constitutes in other species. Despite the in our view fundamentally irreconcilable differences between BETs and the TCE, they provide strikingly similar answers to the question of the current survival value of emotion—although current and evolutionarily adaptive value are confused in a lot of the BET literature. The theories agree that emotions serve to provide the best response to external circumstances to promote the survival and well-being of an individual. This leads us to conclude that the theories, if irreconcilable, can indeed be supplementary in explaining the same complex trait.

To move the field forward, we recommend focusing on what each theory can add to emotion research by using prescriptive definitions (Paul & Mendl, 2018). From this perspective, both BETs and the TCE are important in moving the field of emotion science forward because they can provide answers to questions about different aspects of emotion. The TCE’s focus on the influence of culture, language, and the formation of concepts has shown it is important to be careful applying “folk” labels when studying emotions, especially their evolutionary basis. We support the fundamental view put forward in the TCE that feelings, subjective emotional experiences, are highly variable in similar external circumstances, neurophysiological changes, expressions, and so on. However, we conclude that the evolutionary basis of subjective experiences, especially if language is proposed to play a crucial role, is tightly linked to the evolution of cognition. Conversely, the evolution of emotion focuses on bioregulatory responses to survival- and reproduction-relevant external events. BETs can continue to guide emotion research with a focus on the observable aspects and consequences of these emotions, such as on behavior, (neuro)physiology, expressions, and so on, while carefully describing those aspects and consequences and the situations in which they arise. Such work might result in more support of the BET categories already in use or might reveal different categories at different levels. Furthermore, there might be overlaps in the categories between species (including humans), but there might also be differences.

To elucidate the evolutionary history of emotions, studying emotions in nonhuman animals is crucial. In recent years, animal emotion has started to receive more attention. However, evidence is still limited and mostly focuses on great apes, or in the case of the TCE rhesus macaques. Even in great apes, many open questions remain regarding expressions and (neuro)physiological changes related to emotion, and the subjective experience of emotion has hardly been investigated. For example, further study of (emotional) expressions in carefully described or controlled circumstances can provide insight into subtle differences and similarities in expressions of emotions between great apes and humans. With similar studies in other animals, emotional expressions can be traced further down the phylogenetic tree. Another interesting area of research is ANS activity in response to emotional expressions, emotionally charged scenes, and when exhibiting emotional behavior—specifically again in great apes but also in other animals. This can provide insight into the causation of emotions in animals and possible linked survival value and thus to what might have been adaptive in our evolutionary past. Because the subjective experience of emotions—in ourselves and others—plays an important role in humans, it is also interesting to investigate this in animals. Indeed, by 1997 Burghardt had already proposed a fifth question when studying animal behavior (in addition to Tinbergen’s four) focused on the private experience of animals, including emotion (Burghardt, 1997). Although approaching a best guess of an animal’s internal subjective emotional state is challenging, this can provide insight into the evolutionary history of feeling. As is suggested in the TCE, studies focusing on animals’ cognitive abilities are also interesting in this regard because they can reveal the extent to which animals might be able to process feelings. Finally, it would be interesting to further investigate the proposed interoceptive network in great apes, although this will be challenging because of ethical constraints. In any future work on animal emotions, carefully describing and defining the trait that is under study and how it translates to human emotions is important to be able to interpret the results (Mendl et al., 2022).

Being precise about what aspect of emotion is being studied and using prescriptive definitions rather than descriptive definitions, Tinbergen’s four questions, and perhaps adding a fifth as proposed by Burghardt (1997), remain relevant for studying each aspect and clearly defining and studying their evolution. That way, we can move forward in the study of human and nonhuman animal emotion.

## Transparency

*Action Editor:* Tina M. Lowrey

*Editor:* Interim Editorial Panel

*Author Contributions*

A. Ploeger and K. van Heijst conceptualized the idea for writing the manuscript. K. van Heijst wrote the first draft, and A. Ploeger and M. E. Kret provided feedback. All authors approved the final manuscript for submission.
